# Design Space Approach in Optimization of Fluid Bed Granulation and Tablets Compression Process

**DOI:** 10.1100/2012/185085

**Published:** 2012-07-31

**Authors:** Jelena Djuriš, Djordje Medarević, Marko Krstić, Ivana Vasiljević, Ivana Mašić, Svetlana Ibrić

**Affiliations:** Department of Pharmaceutical Technology, Faculty of Pharmacy, University of Belgrade, 11221 Belgrade, Serbia

## Abstract

The aim of this study was to optimize fluid bed granulation and tablets compression processes using design space approach. Type of diluent, binder concentration, temperature during mixing, granulation and drying, spray rate, and atomization pressure were recognized as critical formulation and process parameters. They were varied in the first set of experiments in order to estimate their influences on critical quality attributes, that is, granules characteristics (size distribution, flowability, bulk density, tapped density, Carr's index, Hausner's ratio, and moisture content) using Plackett-Burman experimental design. Type of diluent and atomization pressure were selected as the most important parameters. In the second set of experiments, design space for process parameters (atomization pressure and compression force) and its influence on tablets characteristics was developed. Percent of paracetamol released and tablets hardness were determined as critical quality attributes. Artificial neural networks (ANNs) were applied in order to determine design space. ANNs models showed that atomization pressure influences mostly on the dissolution profile, whereas compression force affects mainly the tablets hardness. Based on the obtained ANNs models, it is possible to predict tablet hardness and paracetamol release profile for any combination of analyzed factors.

## 1. Introduction

### 1.1. Quality by Design (QbD)

Recently proposed quality-by-design (QbD) regulatory initiative of pharmaceutical product and process development has encouraged researchers in pharmaceutical industry to reach the “desired state” of drug manufacturing in 21st century. Main goal of this approach is to gain a comprehensive understanding of their manufacturing processes, with an accurate estimation of their robustness and reliability. The emphasis has changed from the need to demonstrate that the product will consistently meet relatively tight specifications to a new situation of being able to demonstrate that the product is controlled within a broader “design space” (DS). The design space (DS) concept is introduced as “the multidimensional combination and interaction of input variables (e.g., materials attributes) and process parameters that have been demonstrated to provide assurance of quality.” Using this approach, it is essential to define relationship between critical formulation/process parameters and critical quality attributes (such as granule characteristics and tablet properties) [1, 2].

### 1.2. Fluid Bed Granulation

Wet granulation is a process of small particles agglomeration into larger, relatively permanent structures in which the original particles can still be identified [3]. In fluid bed granulation process, binder solution is usually sprayed in form of the fine droplets onto powder mass in state of fluidization. It is a very complex process influenced by many factors. These factors are classified into three groups: formulation factors, process parameters, and equipment related factors. Their numerous interactions make the process optimization much more difficult [4].

The influence of process parameters and formulation factors on the fluid bed granulation process has been studied extensively in the last few years. The most widely studied formulation factors are type of diluent and binder and concentration of the binder solution. The previous studies proved that, when hydrophilic diluents were used, large-size granules were obtained. This is a result of better wetting of powder mass and consequently faster granules growth [4, 5]. An increase in the binder concentration decreases duration of granulation [6–8]. Higher values of Carr's index and Hausner's ratio, which point to poor flowability, are recorded when higher binder concentration was used [8, 9]. The most extensively studied process parameters are atomization pressure, air flow rate, temperature and humidity of inlet air, and spray rate. It has been shown that increase in the atomization pressure leads to decrease in the binder droplet size [7, 10]. In most studies [9–12], granules size increased with decrease in atomization pressure. Small-size granules were obtained when high air flow rate was used, because of the more intensive breakage and faster evaporation of binder solution [7, 11–13]. Increase in the spray rate leads to the increase in granules size because of the larger droplet size, improved powder mass wetting, and formation of more bonds between the particles [7, 13, 14]. The decrease in granule size was recorded with higher inlet air temperature, because of the faster binder evaporation [11]. Inlet air humidity has the opposite effect; increase in this parameter leads to the increase in granules size as well [15].

### 1.3. Artificial Neural Networks

Artificial neural networks (ANNs) are computer programs designed to simulate some of the human brain functions such as learning, ability to generalize, and draw conclusions from the gained experience. Application of artificial neural networks is a new dimension in the formulation of drugs because of the unique advantages such as nonlinearity, the ability of modeling and optimization with a small set of experiments. ANNs are not programmed, they learn from the presented solved problems. Using different algorithms for learning, they recognize the relationships and patterns within the data presented to them and thus acquire the ability to predict responses to new experimental conditions. The ability of neural networks to generalize the problem is one of the strongest motivations for research in this area [16]. 

**Figure 1:**
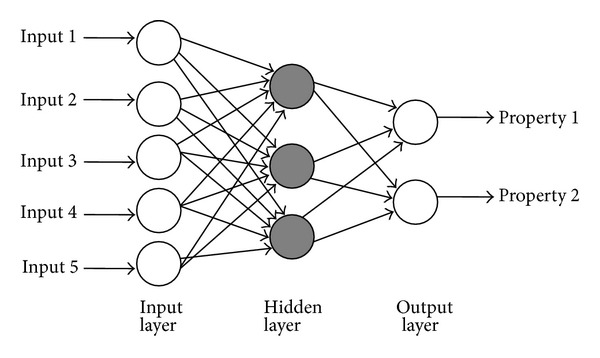
Schematic drawing of multilayer perceptron neural networks [16].

The way in which the neurons are interconnected is referred to as a “network architecture” or “topology.” A variety of network architectures has been developed for different applications, but one of the most common is a multilayer perceptron (MLP) network (Figure 1) in which the neurons are organized into layers. The first layer is called the input layer and has no ability to generate the data. It serves for the transmission of input values to the first hidden layer. The inputs are simply the input variables, such as ingredients, ingredient amounts, and processing conditions. The last layer is used to process the output values of the dependent variables such as in vitro drug release profile and is called the output layer. Between these two layers, there are hidden layers that are used to enable connections between the input and output layers. The complexity of the problem determines the number of the hidden layer neurons. This is a “feed forward” network, which means that information is passed in one direction through the network from the inputs, through various hidden layers to the outputs. If set of inputs variables is provided, the ANNs model can be used to predict the response such as critical quality attribute of the product. Well-trained models can answer the questions like “what if ?” or “what kind of re lease profile can be expected with changing excipients concentration?” [16]. Artificial neural networks can be successfully used in pharmaceutical formulations optimization [17–22].

The aim of our study was to define the design space of fluid bed granulation and tablet compression process. In the first part, the assessment of process and formulation factors (critical material and process parameters) and their influence on granules characteristics (critical quality attributes) was performed. Optimal variables values from the first set of experiments were used in the second set, while atomization pressure and compression force were varied, in order to develop new design space, evaluating their influence on tablets characteristics.

## 2. Materials and Methods

### 2.1. Materials

 Materials used in the presented study for the granulation and tabletting experiments were: paracetamol (Ph.Eur. 7.0), polyethylene oxide (Polyox WSR Coagulant, Dow Chemical, USA, USP30-NF25), anhydrous lactose (Ph.Eur. 7.0), microcrystalline cellulose (Vivapur Type 101, J. Rettenmaier & Söhne GmbH, Germany, Ph.Eur. 7.0), and purified water (Aqua purificata, Ph.Eur. 7.0).

### 2.2. Wet Granulation Process

The granulation was performed in the fluid bed granulator Mycrolab (Hüttlin, Germany). Diluent type, binder (polyethylene-oxide) concentration, temperature during mixing and granulation, air flow rate during mixing, granulation and drying, spray rate, and atomization pressure were varied on two levels (Table 1). Anhydrous lactose or microcrystalline cellulose was used as diluent. Polyethylene-oxide was used as a binder, whereas purified water was used as the binder solvent. For each experimental run, 250 g of granules were prepared, with the granules composition and the processing parameters being given by the number of experimental run, as represented in Table 3. Paracetamol was included in formulations in the second set of experiments. Paracetamol was selected as a highly water-soluble model drug, in order to investigate the possibility to obtain its modified release by using polyethylene oxide polymer as the granules binder. Powder mass was first mixed in the granulator for 5 min. After this, water was sprayed onto the powder mass (containing diluent and polyethylene-oxide) from the bottom of the chamber in the half amount of powder mass. The granulation phase lasted until the whole amount of water was spent. Then, when the product temperature started to rise, the inlet air temperature was set on 0°C in order to cool the product.

### 2.3. Design of Experiments

Plackett-Burman experimental design was used to investigate the influence of formulation and process variables on the granules and tablets characteristics. Coded variables values are given in the Table 2. Experimental plan was obtained after replacing coded with real variables values (Table 3). The result of each experiment is a linear combination of variables effects:

(1) y=β0+β1X1+β2X2+β3X3+β4X4+β5X5  +β6X6+β7X7+ε.

As eight experiments were performed, the system of eight linear equations was obtained. Coefficients *β*1,*β*2,…,*β*7 show how much each of the seven variables influences on granules characteristics. Their values were calculated using *Design Expert*  software (Stat-Ease, USA).

### 2.4. Granules Characterization

#### 2.4.1. Granules Size Distribution

Granules size distribution was determined by the sieve analysis method [23]. A 100-g granule sample was transferred to the set of sieves (180, 315, and 500 *μ*m size) and shaken for 10 min. After 10 minutes of sieving, the fractions of granules retained on each sieve were weighed and these weights were converted into mass percentages.

#### 2.4.2. Flowability

Granules flowability was determined by the official method [23]. A 50 g sample of granules was transferred to the flow testing device (Flow meter GDT, Erweka, Germany), and the time needed for sample flowing through an orifice of 12 mm in diameter was measured. The flowability was expressed as a flow rate which was calculated from the ratio of sample mass and measured time. Results are expressed as the mean value of three replicates.

#### 2.4.3. Bulk Density

Bulk density was determined in the graduated 50 mL cylinder. 15 g of sample was measured and poured into cylinder. The bulk density was calculated as the ratio of granules mass and read volume.

#### 2.4.4. Tapped Density

After determination of the bulk density, graduated cylinder was exposed to agitation and mild striking to the solid surface. The number of taps varied depending on the differences in the granules volume after agitation. Once the volume did not change significantly, striking was stopped. The number of taps did not exceed 100. Tapped density was calculated as the ratio of granules mass and read volume after agitation.

#### 2.4.5. Hausner's Ratio

Hausner's ratio was calculated using the formula:

(2) $$\begin{array}{c} {\text{Hausner}}^{\prime }\text{s}\;\;\text{ratio}=\frac{\text{tapped}\;\;\text{density}}{\text{bulk}\;\;\text{density}}\; \end{array}$$

(see [23]).

#### 2.4.6. Carr's Ratio

Carr's ratio was calculated using the formula

(3) Carr′s  ratio=(trapped  density−bulk  density)trapped  desity

(see [23]).

#### 2.4.7. Moisture Content

The granules moisture content was determined gravimetrically on a Chyo 91 device (Chyo, USA). A certain sample mass was transferred to the device, and after 30 min of warming with the lamp at 105°C, the moisture content was read from the display.

### 2.5. Tablets Preparation

Fluid bed granulation and tabletting of obtained granules on eccentric tablet machine was performed in the second set of experiments. Optimal values of formulation and process factors (X1–X6, Table 1.) were defined by analyzing their influence on granules characteristics in the first set of experiments. Atomization pressure (X7, Table 1) and tablet compression force were varied on three levels in the second set of experiments. Nine formulations (Ft1–Ft9, Table 4) were prepared. The mass of tablets was 500 mg each, while the paracetamol content was 100 mg per tablet.

### 2.6. Tablets Characterization

#### 2.6.1. Resistance to the Crushing of Tablets

This test is intended to determine, under defined conditions, the resistance to crushing of tablets, measured by the force needed to disrupt them by crushing. Resistance to crushing of tablets was determined on Erweka TB24 (Erweka, Germany), measuring the force that leads to the tablet fracture. Results are expressed as the mean value of 10 replicates.

#### 2.6.2. Dissolution Testing

Dissolution testing was performed in the rotating paddle apparatus (Erweka DT70, Germany). Phosphate buffer (pH = 5,8, USP30-NF25) in the volume of 900 mL was used as a medium, and the rotating paddle speed was 50 rpm. Medium temperature was maintained on 37°C to simulate physiological conditions. Sampling was carried out at 5, 10, 20, 30, 45, 60, 90, 120, 150, and 180 minutes, and the absorbance of paracetamol was measured at 243 nm by the UV/VIS spectrophotometer Evolution 300 (Thermo Fisher Scientific, Cambridge, UK). Results are expressed as the mean value of three replicates.

#### 2.6.3. Analysis of Results Using Artificial Neural Networks

Inform^®^ software (Intelligensys, UK) was used to analyze the results of the second set of experiments. The input parameters (independent variables) were atomization pressure and compression force. Tablets hardness and percentage of paracetamol released after 5, 10, 20, 30, 45, 60, 90, 150 and 180 minutes were determined as outputs (dependent variables). ANN used in the study was of the multilayer perceptron (MLP) type with the backpropagation learning algorithm.

## 3. Results and Discussion

### 3.1. Influence of the Formulation and Process Parameters on Granules Characteristics

Characterization of obtained granules was performed after granulation. Granules size distribution, flowability, bulk and tapped density, Hausner's ratio, Carr's index, and granules moisture content were determined as product characteristics. The results of the first set of experiments are given in the Table 5.

The aim was to obtain uniform granules of medium size. The target size was between 180 and 500 *μ*m. Granules smaller than 180 *μ*m tend to have greater cohesiveness, higher Hausner's index, and lower compressibility. Therefore, high percent of this fraction is not desired. The purpose of granulation is to improve the flow properties of powder mix, so high flow rates are desired. As it can be seen from the Table 5, flow rates were very variable. Granules obtained from experiment three were not flowable. This experiment was not successful because the powder was overwetted. Earlier study [11] showed that combination oflow air flow rate, low inlet air temperature, and high spray rate, such as in this experiment, is associated with high risk of overwetting and defluidization. Also, hydrophobic substances such as microcrystalline cellulose are more susceptible to overwetting than hydrophilic ones like anhydrous lactose. Hausner's ratio lower than 1.34 and Carr's index lower than 25% were considered to be acceptable [23]. Higher values that indicate poorer flowability and compressibility are recorded in experiments where granules smaller than 180 *μ*m were obtained in high percentage (Table 5). The moisture content values less than 3.5% were considered to be acceptable. Except experiments 2 and 3, all experiments gave granules with acceptable moisture content.

The analysis of influence of formulation and process factors on granules properties is shown in the Table 6. The diluent type has the strongest influence on each examined characteristic. The fraction of granules with size between 180 and 500 *μ*m increases when anhydrous lactose is selected as a diluent. Granules smaller than 180 *μ*m were obtained in high percentage using microcrystalline cellulose as diluent. This is in concordance with earlier results, which showed that larger granules were obtained when hydrophilic diluents were used [5, 6]. Better flowability and lower moisture content also support the choice of anhydrous lactose as a diluent. The second significant factor is atomization pressure. Larger granules were obtained when lower atomization pressure was used in the process. The same results are recorded in earlier studies [9–12]. But, unexpectedly, granules flowability was not improved with decrease in the atomization pressure. This may be explained with higher moisture content at lower atomization pressure, which increases cohesiveness, and may decrease flowability. For better evaluation of the impact of atomization pressure, this parameter was varied on three levels in the second set of experiments. The temperature during mixing and granulation has the significant influence only on the Carr's index and Hausner's ratio. Higher values of these parameters, which indicate to poor flowability and compressibility, are recorded at higher temperature which is in agreement with earlier results [9]. Other process parameters have less significant influence on granules properties. After analysis of factors influence on the granulation process, process and formulation variables that gave granules with desired properties were chosen: anhydrous lactose as a diluent, higher polyethylene-oxide concentration (10%), lower temperature during mixing and granulation (55°C), lower air flow rate during mixing and granulation (20 m^3^/h), lower air flow rate during drying (20 m^3^/h), higher spray rate (10 g/min). These values were used in the second set of experiments, while atomization pressure was varied on three levels.

### 3.2. Results of the Second Set of Experiments

#### 3.2.1. Tablets Hardness Testing

Results of tablets hardness testing are given in the Table 7. According to results, tablets hardness is very variable, in the range of 3.68 N to 118.21 N, which could have influence on drug release and physical stability of tablets as well.

#### 3.2.2. Drug Release Profiles from Tablets

**Figure 2:**
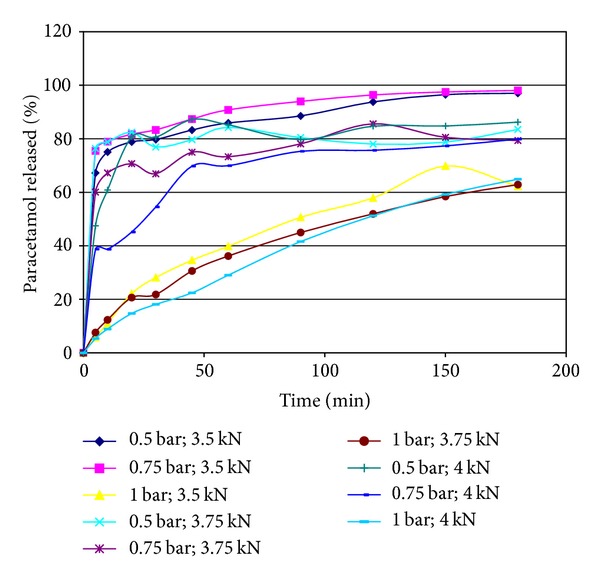
Dissolution profiles of paracetamol from nine tablets series.

Percentages of released paracetamol in different time intervals during 3 hours are given in Table 8. Dissolution profiles are very different (Figure 2). Formulations Ft1, Ft2, Ft4, and Ft7 showed immediate release (after 30 min, over 80% of paracetamol has been released), while formulations where atomization pressure during granulation was 1 bar showed modified release profiles. Percentage of released paracetamol after 30 min varied from 18.12% (Ft9) to 83.30% (Ft2).

#### 3.2.3. Analysis of the Influence of Atomization Pressure and Compression Force on Tablets Hardness Using ANNs

**Figure 3:**
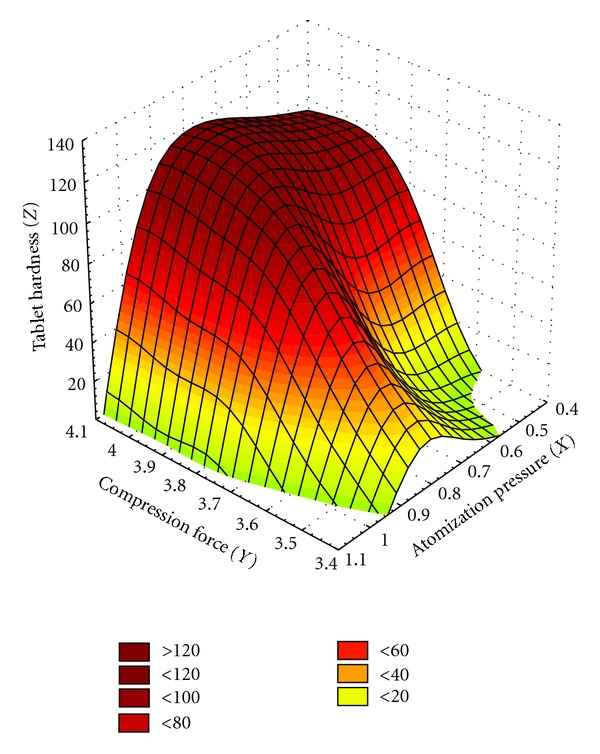
Tablets hardness changing in function of independent variables (atomization pressure and compression force).

Using ANNs, it is possible to visualize dependent variables (tablets hardness and percentage of released paracetamol in different periods of time during 3 h) in function of independent variables (atomization pressure and compression force) by 3D diagrams. It is possible to predict responses in all points of experimental field, because the software finds “ideal” formulation, and further experiments are not necessary.  Figure 3 shows changing of tablets hardness in function of independent variables (atomization pressure and compression force).

3D diagram from Figure 3 shows that atomization pressure has less influence on tablets hardness in comparison to the compression force used for tablet manufacturing. Also, increasing compression force, tablets hardness increased as well.

#### 3.2.4. Analysis of the Influence of Atomization Pressure and Compression Force on Paracetamol Release from Tablets Using ANNs

**Figure 4:**
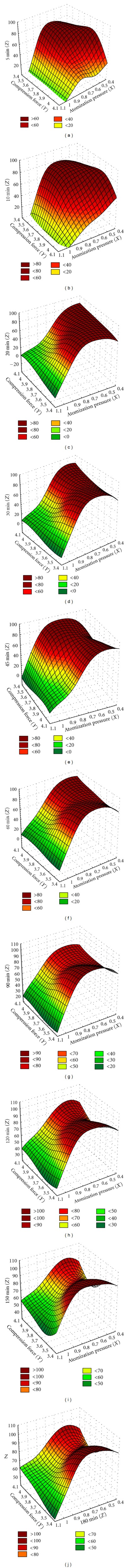
Percent of released paracetamol after 5 (a), 10 (b), 20 (c), 30 (d), 45 (e), 60 (f), 90 (g), 120 (h), 150 (i), and 180 (j) minutes in function of independent variables (atomization pressure and compression force).

Figure 4 presents 3D diagrams which describe the influence of compression force and atomization pressure on paracetamol release. It is clearly seen that both of parameters have influence on release profile, but the impact of atomization pressure is much stronger. The percent of released paracetamol decrease with increasing of atomization pressure and compression force. From obtained results, it may be concluded that high atomization pressure (1 bar) is a good way to achieve modified release of paracetamol from tablets, while increasing of compression force may further support this aim.

**Figure 5:**
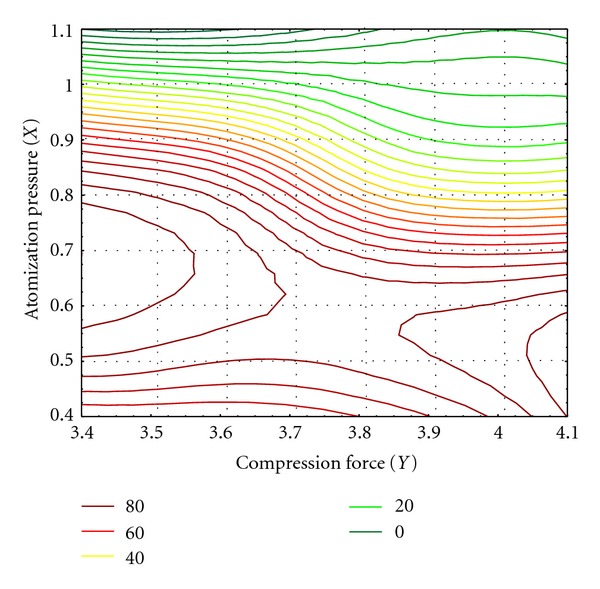
Contour plot which describes the influence of atomization pressure and compression force on the percent of released paracetamol after 30 min.

The main advantage of these models is the possibility to predict drug release and tablets hardness for any combination of atomization pressure and compression force in each point of experimental field.  Figure 5 shows the contour plot describing the influence of atomization pressure and compression force on the percent of paracetamol released after 30 min. It is obvious, that, in wide range of process parameters, the percent of released paracetamol is less than 80%. This shows that polyethylene-oxide delayed release of active substance, which is its purpose in hydrophilic matrix systems.

#### 3.2.5. Predicting Independent Variables (Compression Force and Atomization Pressure) Based on Desired Values of Tablet Hardness and Percentage of Released Paracetamol in Different Time Periods during 3 h Using ANNs

Besides potentially predicting output parameters, it is possible to predict input parameters based on assigned output parameters. First, the information of desired output parameters is being given to the network, and then the network defines their real values and predicts optimal values of input parameters.

The range between minimum and maximum values of desired output parameters is shown in the Table 9. Based on these desired values, defined by the user, network predicts optimal values. In the end, ANN provides the optimal values of input parameters for achieving the desired outputs as in this case atomization pressure of 1 bar and tabletting compression force of 3.66 kN.

## 4. Conclusion

Based on obtained ANNs models, it is possible to predict tablet hardness and released profile of paracetamol for any combination of analyzed factors. This defines design space for fluid bed wet granulation process with paracetamol as an active substance, polyethylene-oxide as a binder, and also design space of obtained granules tabletting process. Quality-by-design approach provides complete knowledge of the process, process control, and observing the principles that “the quality is not being tested, it is built into the product.”

With the development of new user-friendly software package, the growing use of ANNs in design and development of new medicinal preparations is expected. This will also enable the quick and easy evaluation of stability, safety, and efficacy of drugs, with greatly costs reducing.

## Figures and Tables

**Table 1:** Dependent variables in the first set of experiments.

Factor (variable)	Symbol	Low level (−1)	High level (+1)
Type of diluent	X1	Anhydrous lactose	Microcrystalline cellulose
Concentration PEO^1^	X2	5	10
Temperature during mixing and granulation (°C)	X3	55	65
Air flow rate during mixing (m^3^/h)	X4	20	30
Air flow rate during drying (m^3^/h)	X5	20	30
Spray rate (g/min)	X6	5	10
Atomization pressure (bar)	X7	Spray air 0.5	Spray air 1.0
		Microclimate 0.25	Microclimate 0.5

^1^PEO: polyethylene-oxide.

**Table 2:** *Plackett-Burman* experimental design (coded values).

No. exp.	X1	X2	X3	X4	X5	X6	X7
1	−1	−1	−1	+1	−1	+1	+1
2	+1	−1	−1	−1	+1	−1	+1
3	+1	+1	−1	−1	−1	+1	−1
4	−1	+1	+1	−1	−1	−1	+1
5	+1	−1	+1	+1	−1	−1	−1
6	−1	+1	−1	+1	+1	−1	−1
7	−1	−1	+1	−1	+1	+1	−1
8	+1	+1	+1	+1	+1	+1	+1

**Table 3:** Experimental plan for the first set of experiments.

No. exp.	X1	X2 (%)	X3 (°C)	X4 (m^3^/h)	X5 (m^3^/h)	X6 (g/min)	X7 (bar)
1	Anhydrous lactose	5	55	30	20	10	1.0/0.5
2	Microcrystalline cellulose	5	55	20	30	5	1.0/0.5
3	Microcrystalline cellulose	10	55	20	20	10	0.5/0.25
4	Anhydrous lactose	10	65	20	20	5	1.0/0.5
5	Microcrystalline cellulose	5	65	30	20	5	0.5/0.25
6	Anhydrous lactose	10	55	30	30	5	0.5/0.25
7	Anhydrous lactose	5	65	20	30	10	0.5/0.25
8	Microcrystalline cellulose	10	65	30	30	10	1.0/0.5

X1: type of diluent.

X2: binder (polyethylene-oxide) concentration (%).

X3: temperature during mixing and granulation (°C).

X4: air flow rate during mixing (m^3^/h).

X5: air flow rate during drying (m^3^/h).

X6: spray rate (g/min).

X7: atomization pressure (bar).

**Table 4:** Experimental plan for the second set of experiments.

Formulation	Atomization pressure, spray air (bar)	Compression force (kN)
Ft1	0.5	3.5
Ft2	0.75	3.5
Ft3	1.0	3.5
Ft4	0.5	3.75
Ft5	0.75	3.75
Ft6	1.0	3.75
Ft7	0.5	4.0
Ft8	0.75	4.0
Ft9	1.0	4.0

**Table 5:** Granules characterization.

		Y1			Y2	Y3	Y4	Y5	Y6	Y7
	*d* < 180	180 < *d* < 315	315 < *d* < 500	*d* > 500						
1	52.82	39.75	7.38	0.05	7.28 ± 0.6	0.625	0.750	1.20	16.67	1.4
2	97.84	1.86	0.30	0.00	2.90 ± 0.2	0.385	0.517	1.34	25.53	3.9
3	74.31	10.28	6.44	8.97	0.00	0.341	0.468	1.37	27.14	10.1
4	52.73	39.57	7.64	0.06	7.54 ± 0.5	0.600	0.750	1.25	20.00	0.9
5	97.38	2.22	0.40	0.00	2.50 ± 0.2	0.366	0.517	1.41	29.21	3.2
6	35.14	47.97	16.64	0.25	6.02 ± 0.4	0.555	0.682	1.23	18.62	1.0
7	17.72	48.04	33.47	0.78	6.55 ± 0.4	0.469	0.600	1.28	21.83	3.0
8	93.39	5.79	0.83	0.00	2.83 ± 0.2	0.395	0.536	1.36	26.31	3.2

Y1: granules size distribution (%).

Y2: flowability (g/s).

Y3: bulk density (g/mL).

Y4: tapped density (g/mL).

Y5: Hausner's ratio.

Y6: Carr's index (%).

Y7: moisture content (%).

**Table 6:** Analysis of inf luence formulation and process factors on granules properties.

	Granules size distribution				Flowability	Bulk density	Tapped density	Hausner ratio	Carr's index	Moisture content
	<180	180–315	315–500	>500						
Type of diluent (*β*1)	25.5638	−19.3975	−7.145	0.9788	−2.84	−0.095	−0.09125	0.065	3.9163	1.7625
Concentration of PEO (*β*2)	−1.2737	1.4675	−1.25	1.05625	−0.355	0.00525	0.00525	−0.0025	−0.1563	0.4625
Temperature during mixing and granulation (*β*3)	0.1388	−0.53	1.4475	−1.0538	0.4025	−0.0075	−0.00125	0.02	1.1588	−0.7625
Air flow rate during mixing (*β*4)	4.5163	−0.5025	2.825	−1.1888	0.205	0.0175	0.01875	−0.005	−0.4888	−1.1375
Air flow rate during drying (*β*5)	−4.1437	1.48	3.6725	−1.0063	0.1225	−0.0175	−0.01875	−0.0025	−0.0937	−0.0563
Spray rate (*β*6)	−5.6062	1.53	−2.8925	1.1863	0.2875	−0.01	−0.01375	−0.0025	−0.1738	1.0875
Atomization pressure (*β*7)	9.0288	−2.6925	−5.1	−1.2363	0.685	0.0325	0.03625	−0.0175	−1.0513	−0.9875

**Table 7:** Results of tablets hardness testing.

	Compression force 3.5 kN			Compression force 3.75 kN			Compression force 4 kN		
Atomization pressure	0.5 bar	0.75 bar	1 bar	0.5 bar	0.75 bar	1 bar	0.5 bar	0.75 bar	1 bar
Hardness (N)	3.68	26.09	5.59	68.57	84.37	47.48	100.75	118.21	53.96

**Table 8:** The percentage of paracetamol released from tablets in different time interval during 3 hours.

	Compression force 3.5 kN			Compression force 3.75 kN			Compression force 4 kN		
Time (min)	A.P. 0.5 bar	A.P. 0.75 bar	A.P. 1 bar	A.P. 0.5 bar	A.P. 0.75 bar	A.P. 1 bar	A.P. 0.5 bar	A.P. 0.75 bar	A.P. 1 bar
5	67.21 ± 1.94	75.47 ± 3.52	5.81 ± 0.93	76.35 ± 5.18	60.06 ± 9.26	7.61 ± 1.96	47.48 ± 4.23	38.74 ± 1.66	5.37 ± 0.15
10	75.05 ± 2.01	78.91 ± 6.75	11.04 ± 3.19	78.77 ± 4.39	67.20 ± 4.42	12.35 ± 2.87	60.83 ± 5.01	38.71 ± 5.20	8.91 ± 0.11
20	78.83 ± 0.23	81.68 ± 3.82	22.11 ± 8.94	82.39 ± 11.27	70.67 ± 4.53	20.67 ± 6.97	80.68 ± 7.25	45.16 ± 13.08	14.74 ± 0.34
30	79.70 ± 5.90	83.30 ± 3.28	28.13 ± 6.86	76.92 ± 3.57	66.91 ± 5.32	21.81 ± 9.07	80.59 ± 6.05	54.54 ± 16.07	18.12 ± 1.88
45	83.25 ± 1.21	87.34 ± 3.35	34.62 ± 11.67	79.67 ± 2.69	74.96 ± 2.56	30.61 ± 13.00	87.18 ± 2.35	69.79 ± 15.05	22.41 ± 1.45
60	85.87 ± 1.22	90.77 ± 1.46	39.80 ± 11.24	84.21 ± 4.40	73.26 ± 7.55	36.18 ± 10.72	85.20 ± 3.84	69.93 ± 14.56	29.03 ± 1.50
90	88.50 ± 1.96	93.93 ± 2.02	50.65 ± 12.73	80.43 ± 11.71	78.09 ± 6.81	44.98 ± 12.02	79.69 ± 5.56	75.31 ± 10.91	41.61 ± 1.21
120	93.75 ± 0.55	96.35 ± 0.02	57.85 ± 12.54	77.99 ± 2.56	85.56 ± 8.07	51.94 ± 12.02	84.69 ± 9.95	75.68 ± 10.67	51.22 ± 0.19
150	96.46 ± 2.53	97.46 ± 3.08	69.80 ± 12.22	78.73 ± 4.96	80.51 ± 3.10	58.44 ± 10.66	84.75 ± 4.89	77.31 ± 7.31	59.24 ± 0.88
180	97.00 ± 1.88	98.00 ± 2.95	62.25 ± 8.66	83.49 ± 8.60	79.33 ± 9.33	62.87 ± 10.00	86.19 ± 6.33	79.86 ± 7.61	64.89 ± 1.71

A.P.: atomization pressure.

**Table 9:** The prediction of input parameters based on desired values of outputs.

	Desired output parameters		Predicted parameters
	Min	Max
Hardness (N)	60	80	61.83

Percent of released paracetamol in different time intervals (%)			

5 min	5	10	15.99
10 min	10	15	16.55
20 min	15	20	18.83
30 min	20	25	23.67
45 min	25	35	30.39
60 min	35	45	38.23
90 min	45	55	49.98
120 min	55	65	59.85
150 min	65	80	67.82
180 min	80	90	63.34
